# Exogenous LIN28 Is Required for the Maintenance of Self-Renewal and Pluripotency in Presumptive Porcine-Induced Pluripotent Stem Cells

**DOI:** 10.3389/fcell.2021.709286

**Published:** 2021-07-20

**Authors:** Warunya Chakritbudsabong, Somjit Chaiwattanarungruengpaisan, Ladawan Sariya, Sirikron Pamonsupornvichit, Joao N. Ferreira, Panithi Sukho, Dulyatad Gronsang, Theerawat Tharasanit, Andras Dinnyes, Sasitorn Rungarunlert

**Affiliations:** ^1^Laboratory of Cellular Biomedicine and Veterinary Medicine, Faculty of Veterinary Science, Mahidol University, Nakhon Pathom, Thailand; ^2^Department of Clinical Sciences and Public Health, Faculty of Veterinary Science, Mahidol University, Nakhon Pathom, Thailand; ^3^Department of Preclinic and Applied Animal Science, Faculty of Veterinary Science, Mahidol University, Nakhon Pathom, Thailand; ^4^The Monitoring and Surveillance Center for Zoonotic Diseases in Wildlife and Exotic Animals (MOZWE), Faculty of Veterinary Science, Mahidol University, Nakhon Pathom, Thailand; ^5^Exocrine Gland Biology and Regeneration Research Group, Faculty of Dentistry, Chulalongkorn University, Bangkok, Thailand; ^6^Department of Obstetrics, Gynecology and Reproduction, Faculty of Veterinary Science, Chulalongkorn University, Bangkok, Thailand; ^7^BioTalentum Ltd., Gödöllő, Hungary; ^8^Department of Physiology and Animal Health, Institute of Physiology and Animal Health, Hungarian University of Agriculture and Life Sciences, Gödöllő, Hungary; ^9^College of Life Sciences, Sichuan University, Chengdu, China

**Keywords:** LIN28, reprogramming, induced pluripotent stem cells, porcine, self-renewal, pluripotency, differentiation, cardiomyocytes

## Abstract

Porcine species have been used in preclinical transplantation models for assessing the efficiency and safety of transplants before their application in human trials. Porcine-induced pluripotent stem cells (piPSCs) are traditionally established using four transcription factors (4TF): OCT4, SOX2, KLF4, and C-MYC. However, the inefficiencies in the reprogramming of piPSCs and the maintenance of their self-renewal and pluripotency remain challenges to be resolved. LIN28 was demonstrated to play a vital role in the induction of pluripotency in humans. To investigate whether this factor is similarly required by piPSCs, the effects of adding LIN28 to the 4TF induction method (5F approach) on the efficiency of piPSC reprogramming and maintenance of self-renewal and pluripotency were examined. Using a retroviral vector, porcine fetal fibroblasts were transfected with human OCT4, SOX2, KLF4, and C-MYC with or without LIN28. The colony morphology and chromosomal stability of these piPSC lines were examined and their pluripotency properties were characterized by investigating both their expression of pluripotency-associated genes and proteins and *in vitro* and *in vivo* differentiation capabilities. Alkaline phosphatase assay revealed the reprogramming efficiencies to be 0.33 and 0.17% for the 4TF and 5TF approaches, respectively, but the maintenance of self-renewal and pluripotency until passage 40 was 6.67 and 100%, respectively. Most of the 4TF-piPSC colonies were flat in shape, showed weak positivity for alkaline phosphatase, and expressed a significantly high level of SSEA-4 protein, except for one cell line (VSMUi001-A) whose properties were similar to those of the 5TF-piPSCs; that is, tightly packed and dome-like in shape, markedly positive for alkaline phosphatase, and expressing endogenous pluripotency genes (*pOCT4, pSOX2, pNANOG*, and *pLIN28*), significantly high levels of pluripotent proteins (OCT4, SOX2, NANOG, LIN28, and SSEA-1), and a significantly low level of SSEA-4 protein. VSMUi001-A and all 5F-piPSC lines formed embryoid bodies, underwent spontaneous cardiogenic differentiation with cardiac beating, expressed cardiomyocyte markers, and developed teratomas. In conclusion, in addition to the 4TF, LIN28 is required for the effective induction of piPSCs and the maintenance of their long-term self-renewal and pluripotency toward the development of all germ layers. These piPSCs have the potential applicability for veterinary science.

## Introduction

The development of genetic reprogramming tools for generating induced pluripotent stem cells (iPSCs) from somatic cells is a promising strategy in regenerative medicine. The iPSC cultures can provide limitless sources of cells for biomedical research, disease modeling, drug discovery and screening, toxicity testing, and patient-specific cell transplantation (Takahashi et al., 2007; Yu et al., 2007; Singh et al., 2015). However, iPSC transplants must undergo various stages of animal testing of their efficiency and safety before they can be applied to humans. Because *Sus scrofa* species (domestic pigs) have similar anatomical, physiological, and immunological attributes to humans (Hall, 2008; Groenen et al., 2012; Moradi et al., 2019), they have been widely used as test models in preclinical transplantation medicine (Harding et al., 2013) and especially in myocardial therapy (Li et al., 2013). Moreover, piPSCs would produce available cell resources to study embryonic development and cell differentiation of these species for screening and establishing desired traits for sustainable agricultural production for veterinary medicine. Therefore, piPSCs are innovative therapies for veterinary medicine (Su et al., 2020).

Porcine iPSCs (piPSCs) are typically generated using both viral-based integration and non-integration methods. Although most piPSCs are established using four transcription factors (4TF)–octamer-binding transcription factor 4 (OCT4), SRY-box transcription factor 2 (SOX2), Kruppel-like factor 4 (KLF4), and MYC proto-oncogene, basic helix–loop–helix transcription factor (C-MYC)–which are introduced *via* retroviral vector transduction, the reprogramming efficiency is lower than that for mouse iPSCs (miPSCs) and human iPSCs (hiPSCs) (Esteban et al., 2009; Ezashi et al., 2009; Wu et al., 2009). It was previously found that hiPSCs could be efficiently reprogrammed using a viral method expressing the 4TF OCT4, SOX2, Lin-28 (LIN28), and Nanog homeobox (NANOG) (Yu et al., 2007). Subsequently, Tanabe et al. (2013) combined LIN28 with the 4TF approach for generating hiPSCs and reported a significant increase in the number of colonies produced and that LIN28 had supported complete cell reprogramming. The endogenous LIN28 was activated later during the reprogramming of the hiPSCs while maturation was taking place and therefore also improved the maturation of the cells (Tanabe et al., 2013). Aside from improving hiPSC colony formation, LIN28 overexpression could enhance the efficiency of hiPSC derivation. Conversely, the depletion of endogenous LIN28 decreased the efficiency of miPSC reprogramming (Zhang J. et al., 2016). Moreover, LIN28 promoted high reprogramming efficiency during miPSC generation by inducing an increase in the rate of cell division (Hanna et al., 2009).

LIN28, an RNA-binding protein found in nucleolus precursor bodies during embryogenesis (Vogt et al., 2012), has two paralogs: LIN28A and LIN28B. The expression of LIN28 in an embryo is restricted to some differentiated cells, such as cardiomyocytes, epithelial cells of the lung and kidney, and neuroepithelial cells (Yang and Moss, 2003). In adult cells, LIN28 remains expressed in kidney epithelial cells, cardiomyocytes, skeletal myocytes, and red blood cells (Tsialikas and Romer-Seibert, 2015). LIN28 plays an essential role in both embryonic stem cell (ESC) and iPSC self-renewal and also promotes the number of ESCs and their proliferation (Xu et al., 2009). When LIN28 is highly upregulated, it binds to both pri- and pre-*let-7* microRNAs, thereby inhibiting the maturation of *let-7* and restraining the differentiation of iPSCs (Shyh-Chang and Daley, 2013). When mouse ESCs differentiate, the expression of LIN28 is downregulated. It was demonstrated that LIN28 could regulate glucose and amino acid metabolism in both ESCs and iPSCs, where the lack of LIN28 decreased nucleotide and glucose metabolism in the stem cells and inhibited their proliferation (Nguyen and Zhu, 2015).

LIN28 has already been used together with core embryonic TFs to generate miPSCs and hiPSCs. In 2017, our group registered the establishment of one piPSC line reprogrammed from porcine fetal fibroblasts (PFFs) with the addition of LIN28 to OSKM (hOCT4, hSOX2, hKLF4, and hC-MYC) TF (Chakritbudsabong et al., 2017). However, no comparison was performed to understand the reprogramming advantages of 5TF (OSKM with Lin28) over 4TF (OSKM without Lin28). Hence, a comparative study is deemed necessary. This current study was carried out to investigate the effects of LIN28 addition to the traditional 4TF on the efficiencies of piPSC generation and reprogramming. Additionally, the biological effects of LIN28 on piPSC self-renewal and pluripotency were evaluated. Our findings provide a solution for improving the induction and reprogramming efficiencies of piPSCs and the maintenance of their self-renewal and pluripotency and allow for the effective scale-up production of piPSC-derived cardiomyocytes for application in research studies on cardiovascular diseases and treatments.

## Materials and Methods

### Ethics Statement

The Institutional Animal Care and Use Committee at the Faculty of Veterinary Science, Mahidol University, Thailand, approved the experimental use of animals (Approval ID: VSMU-2012-57).

### Animals

A male porcine fetus (at embryonic day 28) from a crossbred pig (Large White/Landrace × Duroc) was procured from a certified farm at Ratchaburi Province, Thailand. Pregnant ICR mice at 13–14 days post-coitum were used for the generation of feeder cells, and 6-weeks-old female nude mice (BALB/cAJcl-nu/nu) were used for testing the formation of teratomas. All mice were purchased from Nomura Siam International Co., Ltd., Bangkok, Thailand.

### Reagents

All cell culture reagents and chemical compounds were obtained from Thermo Fisher Scientific (Waltham, MA, United States) and Sigma-Aldrich (St. Louis, MO, United States), respectively, unless otherwise stated.

### Cell Culture

Porcine fetal fibroblasts from the porcine fetus were prepared using standard procedures (Cheng et al., 2012). The GP2-293 cells (a HEK 293-based retroviral packaging cell line), PFFs, and mitomycin C-inactivated mouse embryonic fibroblasts (iMEFs) were maintained in fibroblast medium made up of Dulbecco’s modified Eagle’s medium (DMEM)-high glucose supplemented with 10% fetal bovine serum (cat. no. SV30160, Hyclone, Logan, UT, United States), 1% GlutaMAX^TM^, and 1% antibiotic-antimycotic solution. The piPSC lines were cultured in piPSC medium made up of DMEM/F-12 supplemented with 10% Knockout^TM^ serum replacement, 10% fetal bovine serum (cat no. SH30070, Hyclone, Logan, UT, United States), 1% GlutaMAX^TM^, 1% antibiotic-antimycotic solution, 0.1 mM non-essential amino acids, 0.1 mM 2-mercaptoethanol, 1,000 U/mL mouse leukemia inhibitory factor (LIF; ESG1107, Millipore, Burlington, MA, United States), and 10 ng/mL human basic fibroblast growth factor (bFGF; 233-FB-025/CF, R&D Systems, Minneapolis, MN, United States). The piPSCs were maintained on iMEF and passaged using 1% TrypLE^TM^ Select every 2 days. The cells were cultured in differentiation medium (piPSC medium without LIF and bFGF) to induce their differentiation into all three germ layers and cardiomyocytes. All cells were incubated in a humidified incubator under 5% CO_2_ at 37°C. The piPSC medium was changed daily, whereas the differentiation medium was changed every 2 days.

### Retroviral Vector Transduction and piPSC Generation

**FIGURE 1:**
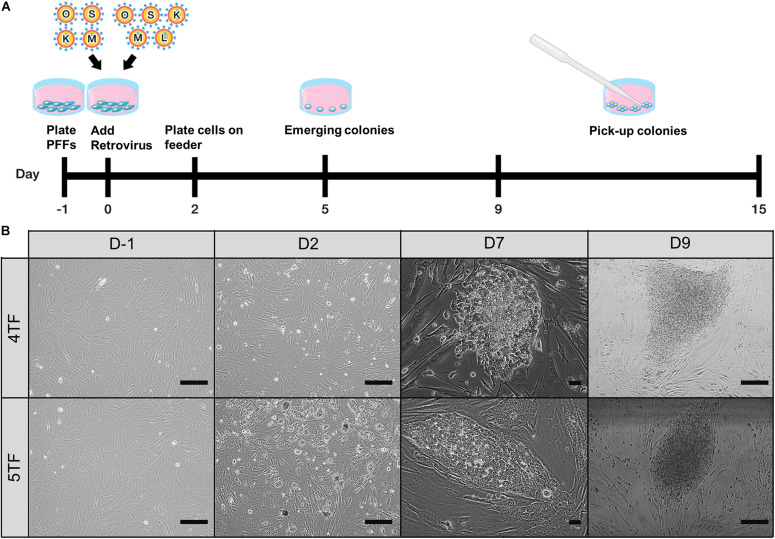
Establishment of porcine-induced pluripotent stem cells (piPSCs). **(A)** Schematic diagram of the establishment of piPSCs. **(B)** The process of generation of 4TF-piPSC and 5TF-piPSC; scale bar, 50 μm. 4TF, induction using OCT4, SOX2, KLF4, and C-MYC; 5TF, induction using OCT4, SOX2, KLF4, C-MYC, and LIN28.

The retroviral vector transduction and piPSC generation were performed according to the protocols described in a previous report (Esteban et al., 2009). Two different reprogramming factor combinations were used: 4TF, and 4TF plus LIN28 (i.e., 5TF). In brief, GP2-293 cells were seeded at 2 × 10^6^ cells in 100 mm dishes. After 24 h, pMX plasmids carrying the human monocistronic reprogramming factors (4TF or 5TF) were transfected into the GP2-293 cells using the calcium phosphate transfection protocol. The supernatant with virus particles was collected at 48 and 72 h after transduction, filtered through a 0.45 μm membrane (Millipore), and then directly used to infect the PFFs. At day 2 post-reprogramming, the PFFs were dissociated with 0.25% trypsin-EDTA solution and re-seeded in six-well plates containing iMEFs and piPSC medium. At days 9–15 post-reprogramming, primary colonies with an ESC-like morphology were separated mechanically into small fractions using a Pasteur pipette and transferred to a Falcon^®^ IVF one-well dish with iMEFs (Figure 1A). After the colonies had redeveloped, they were routinely passaged with TrypLE^TM^ Select. The reprogramming efficiency was calculated as the number of AP positive colonies divided by the total number of transfected cells (Setthawong et al., 2019).

### Alkaline Phosphatase and Immunofluorescence Staining

The piPSCs and differentiated cells were fixed with 4% paraformaldehyde in phosphate-buffered saline (PBS) for 15 min and then washed three times with cold PBS. The piPSCs were subjected to the alkaline phosphatase (AP) assay by staining with a Leukocyte AP Kit (86R-1KT) according to the manufacturer’s protocol. The immunofluorescence (IF) assay was used for detecting pluripotency and cardiac differentiation markers. In brief, the fixed cells were incubated with a cell permeabilization solution (0.25% Triton-X 100 in PBS) for 10 min and then incubated with a blocking solution (2% bovine serum albumin) for 1 h. Then, the cells were treated with the primary antibodies at 4°C overnight and subsequently incubated with the secondary antibodies at 37°C for 1 h (see Supplementary Table 1 for the list of primary and secondary antibodies used). The cell nuclei were counterstained with 4′-6-diamidino-2-phenylindole. All cells were visualized using a Leica DMi8 inverted fluorescence microscope, and images were captured with an attached Leica DFC7000 camera (Leica Microsystems, Wetzlar, Germany). At least 30 z-stacks were obtained for each sample, with 0.6–0.7 μm intervals. Leica Application Suite X (LAS X) imaging software was used for the analysis of all images and for the quantitative measurement of the IF signals. The IF intensities of the immunoreactive pixels of OCT4, SOX2, LIN28, NANOG, stage-specific embryonic antigen (SSEA)-1, and SSEA-4 were measured in 20 randomly selected fields per piPSC line under ×400 magnification. At least three slides per group were scanned for the expression of these markers. Data are presented as the mean IF intensity value ± SEM after subtraction of the background signal.

### G-Banding Karyotype Analysis

Karyotype analysis was performed using a previously published protocol, with slight modifications (Phakdeedindan et al., 2019). In brief, the various piPSCs (passage 20) were incubated with 5 μg/mL colcemid (KaryoMAX^TM^ Colcemid^TM^ Solution in PBS) at 37°C for 1 h. Then, the cells were dissociated and treated with 75 M KCl at 37°C for 15 min. Thereafter, the cells were fixed three times in a cold fixative solution (1:3 acetic acid:methanol concentration) for 10 min each time with gentle inversion to allow mixing. The fixed cells were then dropped onto cold glass slides and incubated at 37°C overnight. Finally, the cells were observed under a Nikon Eclipse Ni microscope equipped with a DS-Ri2 camera (Nikon Instruments, Tokyo, Japan). In total, 50 G-banded metaphases per piPSC culture were analyzed using the LUCIA Cytogenetics System (Nikon Instruments).

### Spontaneous Cardiogenic Differentiation of the piPSCs via Embryoid Body Formation

Spontaneous cardiogenic differentiation of the piPSCs was induced as described in a previously published report (Rungarunlert et al., 2013). In brief, floating embryoid bodies (EBs) were formed in the poly(2-hydroxyethyl methacrylate)-coated wells of 96-well plates using 5,000 piPSCs per well and maintained in differentiation medium for 21 days. On days 7 and 21 of culture, the floating EBs were collected for morphological characterization and for determination of their gene expression levels by reverse transcription polymerase chain reaction (RT-PCR) assay. To obtain adherent EBs, the EBs at day 3 were plated onto 0.1% gelatin-covered coverslips placed in the wells of a 24-well plate (1 EB/well) together with differentiation medium. The morphology of the differentiated cells and their cardiac beating were checked daily. Cells were collected at days 7, 14, and 21 for analysis of their gene expression profiles using RT-PCR. Additionally, cells were fixed with 4% paraformaldehyde for IF staining.

### Teratoma Formation

For each piPSC line, two nude mice (8 weeks old) were subcutaneously injected with 8 × 10^6^ cells into the right flank. After 35 days, the mice were euthanized and the teratomas were collected, fixed with PBS containing 10% neutral buffered formalin, and embedded in paraffin. Sections sliced from the paraffin blocks were then stained with hematoxylin and eosin to confirm the capacity of the cells to differentiate into all three embryonic germ layers *in vivo*. Images of the teratomas were captured using an Axioskop 40 microscope equipped with an AxioCam MRc camera (Carl Zeiss, Oberkochen, Germany).

### RT-PCR Analysis of Transgene and Endogenous Gene Expression

The expression of transgenes and endogenous genes by the various piPSCs and differentiated cells was examined by RT-PCR assay. Cells were lysed for RNA extraction using the RNeasy Mini Kit (Genaid Biotech Ltd., New Taipei City, Taiwan). Then, 1 μg of total RNA was reverse transcribed to cDNA using the SuperScript^TM^ III First-Strand Synthesis System. The PCR mixture contained 50 ng of template cDNA, 12.5 μL of GoTaq PCR master mix (Promega, WI, United States), and 0.2 μM of each primer. The PCR-amplified products were separated on 2% agarose gels and then visualized with GelRed^®^ nucleic acid staining (Biotium, Fremont, CA, United States). The primer oligonucleotide sequences are shown in Supplementary Table 2.

### Capillary Western Blot Analysis

Cell samples were lysed by sonication in radioimmunoprecipitation assay buffer and the protein quantity was then detected using a protein assay kit (Bio-Rad Laboratory, Hercules, CA, United States). Next, capillary western blot analysis of the proteins was performed in 25 capillary cartridges according to the 12–230 kDa Jess and Wes Separation Module protocol (SM-W004, ProteinSimple, San Jose, CA, United States). In brief, a mixture of total protein with a fluorescent dye (4:1 ratio) was heated at 95°C for 5 min. After this denaturation step, the biotinylated ladder, protein sample, blocking reagent, primary antibodies (Supplementary Table 1), horseradish peroxidase-conjugated secondary antibodies, and chemiluminescent substrate were dispensed into a Jess assay plate according to the kit manual instructions. Thereafter, the separation and immunodetection of the proteins were performed with the Jess automated western blotting system (ProteinSimple). The results were analyzed using Compass for Simple Western version 5.0.1 software (Build 0911; ProteinSimple).

### Flow Cytometric Analysis

The piPSCs were harvested and dissociated into single cells using 1% TrypLE^TM^ Select. Thereafter, the cells were incubated with BD Cytofix/Cytoperm^TM^ (BD Biosciences, Franklin Lakes, NJ, United States) at 4°C for 20 min and then washed three times with PBS. A blocking solution (3% bovine serum albumin) was then added and the cells were incubated for 30 min at ambient temperature. Then, the cells were stained with primary antibodies against the various markers at 4°C overnight. After washing with PBS, the cells were stained with fluorescence-labeled secondary antibodies at 37°C for 1 h. All samples were single-color stained, with 20,000 cells used for each marker. Samples incubated with IgG isotype antibodies were used as a negative control. The results were analyzed using FACScalibur and Cell Quest software (BD Biosciences). The list of primary and secondary antibodies used is shown in Supplementary Table 1.

### Statistical Analysis

All experiments were repeated three times. Quantitative data are presented as the mean ± SEM from three independent experiments. One-way analysis of variance was used for the comparison of more than two groups, and Tukey’s test was used as a *post hoc* test. All statistical analyses were performed using SPSS version 25.0 (IBM, Armonk, NY, United States), with statistical significance set at *P* < 0.05.

## Results

### Establishment of the Various piPSC Lines

**FIGURE 2:**
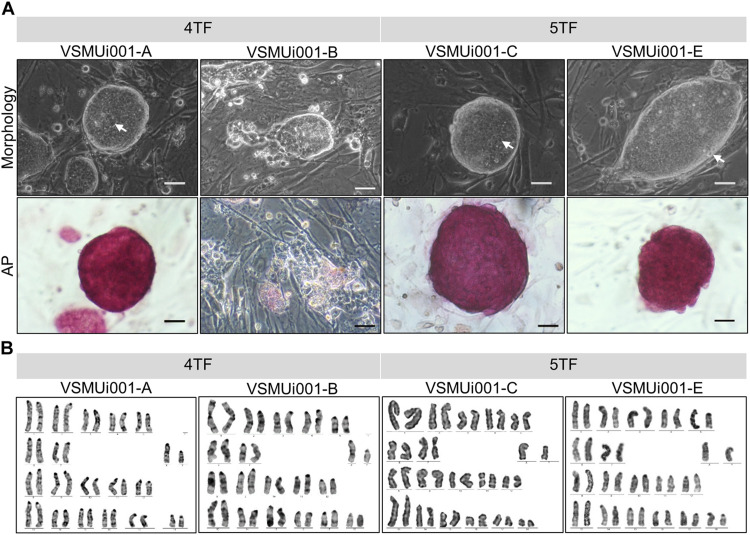
Characterization of piPSCs. **(A)** Colony morphology and alkaline phosphatase (AP) staining of various piPSC lines; scale bar, 20 μm. **(B)** Karyotypes of the various piPSC lines. 4TF, induction using OCT4, SOX2, KLF4, and C-MYC; 5TF, induction using OCT4, SOX2, KLF4, C-MYC, and LIN28. AP, alkaline phosphatase; piPSCs, porcine-induced pluripotent stem cells.

To test the role of LIN28 in cell reprogramming, PFFs were transduced with retroviral vectors designed to express either 4TF or 5TF. The schematic diagram of the establishment of the piPSCs is shown in Figure 1A. The ESC-like colonies generated through the 4TF and 5TF approaches were first observed on day 9 after retroviral transduction. On day 15 after transduction, the colonies were picked and mechanically passaged on iMEFs (Figure 1B). The 4TF− and 5TF-induced clones were designated as 4TF-piPSCs and 5TF-piPSCs, respectively. AP staining of the cells revealed the reprogramming efficiency (i.e., as reflected by the percentage of AP-positive colonies) of 4TF to be 0.33% and that of 5TF to be 0.17%. We obtained approximately fifteen 4TF-piPSC-like colonies from 4,500 initial transfected cells and three 5TF-piPSC-like colonies from 1,800 initial transfected cells (Supplementary Table 3). The percentage of cells maintaining self-renewal and pluripotency until passage 20 was 13.33% with the 4TF system but increased to 100% with 5TF induction. Moreover, the percentage of cells maintaining self-renewal and pluripotency until passage 40 increased from 6.67% with the 4TF system to 100% with 5TF induction. The induced day of 4TF and 5TF system was 11.5 ± 2.65 and 12 ± 3, respectively. In total, we generated three 5TF-piPSC and two 4TF-piPSC lines. We selected only two cell lines from each induction group based on their unlimited self-renewal ability and pluripotency (AP positive staining), namely, VSMUi001-A and VSMUi001-B from the 4TF group and VSMUi001-C and VSMUi001-E from the 5TF group as observed in Figure 2A. The discarded cell lines with limited self-renewal ability and pluripotency are shown in Supplementary Figure 1 and on our previous publication (Chakritbudsabong et al., 2017).

### Morphology and Proliferation of the piPSCs

Except for VSMUi001-A, all the 4TF-piPSC lines had flat-shaped colonies. By contrast, the VSMUi001-A colonies were tightly packed and dome-like in shape with clear edges and were indistinguishable from the 5TF-piPSC lines and mouse ESCs in terms of morphology. Moreover, the cells had a high nuclear-to-cytoplasm ratio, with dominant nucleoli (Figure 2A). VSMUi001-A and all the 5TF-piPSC lines were strongly positive for AP staining (Figure 2A), whereas the rest of the 4TF-piPSC lines were weakly positive in this regard. The proliferative activity of the piPSC lines was determined from their population doubling times, which were calculated by quantifying the cell count at every 12 h over a 48 h period. The VSMUi001-C cell line had the lowest population doubling time (∼9–10 h) compared with all the other piPSC lines (∼12 h) (Supplementary Figure 2). Moreover, only the VSMUi001-A and 5TF-piPSC lines could be passaged continuously for more than 40 passages (a maximum passages) as followed: VSMUi001-A (P47), VSMUi001-C (P42), and VSMUi001-E (P42). By contrast, all the other 4TF-piPSC lines could not be maintained beyond 20 passages owing to their low expansion rates and morphological changes. Both the 4TF-piPSCs and 5TF-piPSCs carried the normal porcine diploid karyotype (38, XY) throughout the extended culture periods (Figure 2B).

### Pluripotency in the Various piPSC Lines

**FIGURE 3:**
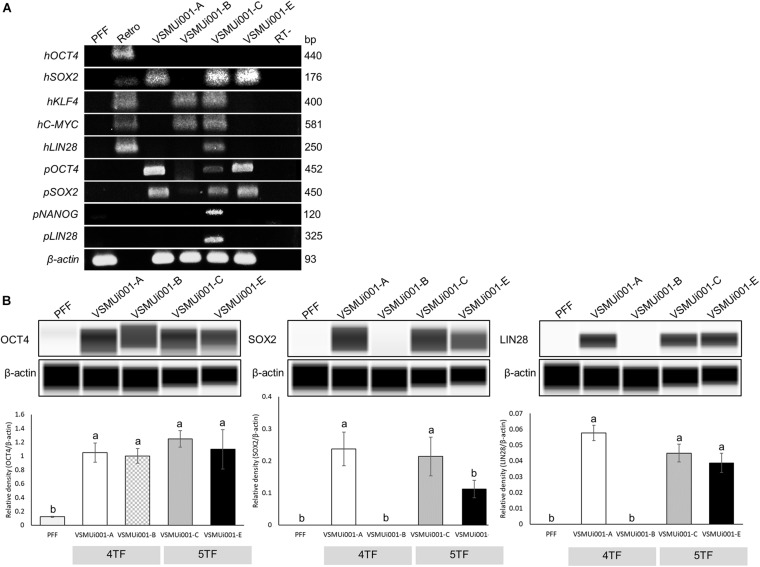
Expression of pluripotency markers in various piPSC lines. **(A)** Expression of exogenous pluripotency genes (*hOCT4*, *hSOX2*, *hKLF4*, *hC-MYC*, and *hLIN28*) and endogenous pluripotency genes (*pOCT4*, *pSOX2*, *pNANOG*, and *pLIN28*) in the PFFs and various piPSC lines. **(B)** Western blot images of endogenous pluripotent protein (OCT4, SOX2, and LIN28) expression and quantification of the western blot results. β-Actin was used as an internal control. Means with different lowercase letters are significantly different at *P* < 0.05. PFF, porcine fetal fibroblast; piPSCs, porcine-induced pluripotent stem cells; *h*, exogenous human gene; *p*, endogenous porcine gene; *OCT4*, octamer-binding transcription factor 4; *SOX2*, SRY-box transcription factor 2; *KLF4*, Kruppel-like factor 4; *C-MYC*, MYC proto-oncogene, basic helix–loop–helix transcription factor; *LIN28*, Lin-28 homolog A; *NANOG*, Nanog homeobox.

**FIGURE 4:**
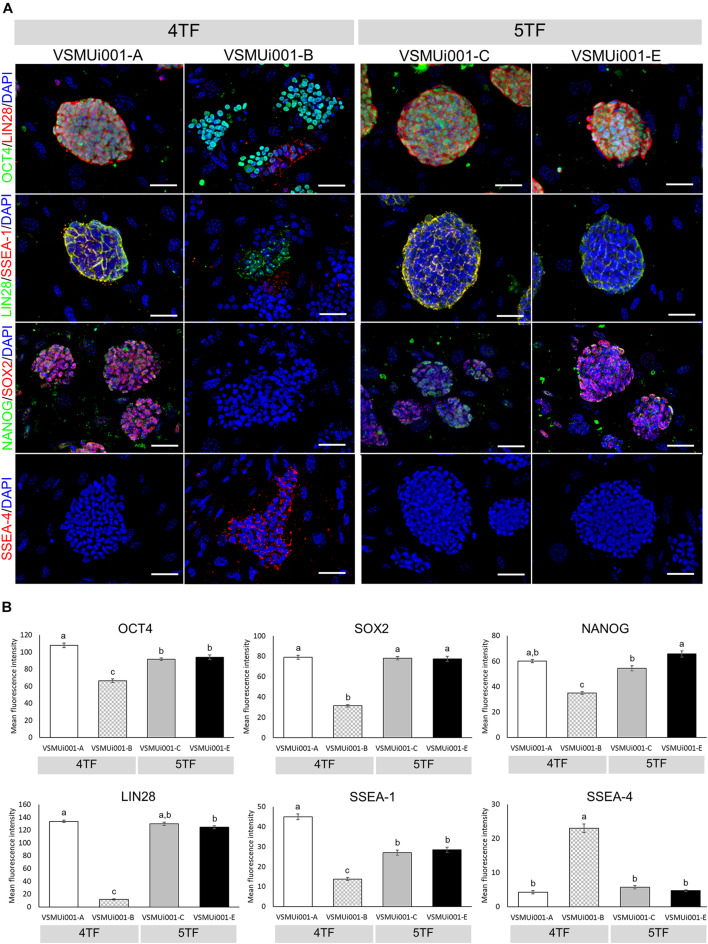
Immunofluorescence analysis of pluripotency markers in various piPSC lines. **(A)** Detection of pluripotency markers in 4TF-piPSC and 5TF-piPSC lines using fluorescence microscopy; scale bar: 50 μm. **(B)** Quantitative analysis of the pluripotency markers in the various piPSC lines. The mean fluorescence signals for OCT4, SOX2, LIN28, NANOG, SSEA-1, and SSEA-4 were measured in 20 randomly selected fields per piPSC colony under identical optical settings. Means with different lowercase letters are significantly different at *P* < 0.05. 4TF, induction using OCT4, SOX2, KLF4, and C-MYC; 5TF, induction using OCT4, SOX2, KLF4, C-MYC, and LIN28. OCT4, octamer-binding transcription factor 4; SOX2, SRY-box transcription factor 2; LIN28, Lin-28 homolog A; NANOG, Nanog homeobox; SSEA, stage-specific embryonic antigen.

**FIGURE 5:**
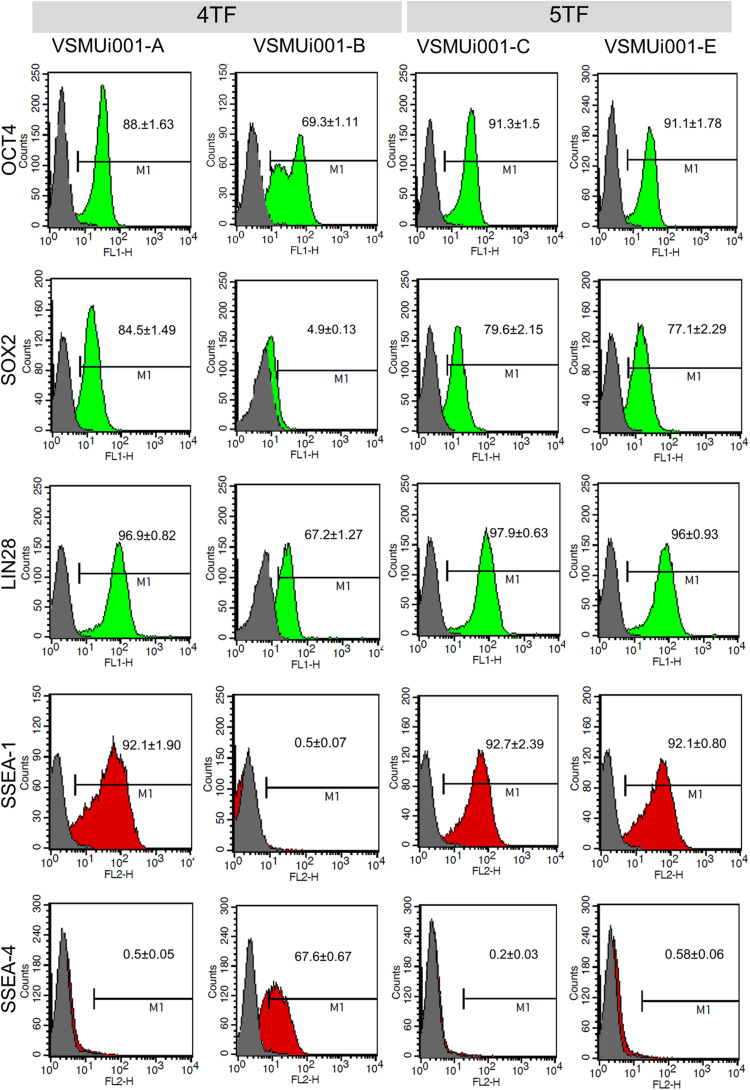
Flow cytometric analysis of pluripotency markers expressed by various porcine-induced pluripotent stem cell lines. 4TF, induction using OCT4, SOX2, KLF4, and C-MYC; 5TF, induction using OCT4, SOX2, KLF4, C-MYC, and LIN28. OCT4, octamer-binding transcription factor 4; SOX2, SRY-box transcription factor 2; LIN28, Lin-28 homolog A; SSEA, stage-specific embryonic antigen.

Reverse transcription polymerase chain reaction analysis confirmed the re-activation of the human exogenous genes and the activation of the porcine endogenous genes in the piPSCs. The 4TF-piPSCs displayed high expression levels of the endogenous pluripotency-associated transcription factors *pOCT4*, *pSOX2*, and *pNANOG*, whereas the 5TF-piPSCs expressed *pOCT4*, *pSOX2*, *pNANOG*, and *pLIN28* at a high level. By contrast, the PFFs and parent cells did not express any of the human exogenous and porcine endogenous pluripotency-associated genes (Figure 3A, Supplementary Figure 3). To confirm the RT-PCR data, capillary western blot analysis was performed to detect the endogenous OCT4, SOX2, and LIN28 proteins. Quantitative analysis of the protein levels relative to that of β-actin (an internal control) revealed that the expression of OCT4 was similar in the 4TF-piPSC and 5TF-piPSC lines (1.1 ± 0.14, 1 ± 0.11, 1.3 ± 0.12, and 1.1 ± 0.29 in VSMUi001-A, VSMUi001-B, VSMUi001-C, and VSMUi001-E, respectively). Moreover, in VSMUi001-A, VSMUi001-C, and VSMUi001-E, the expression levels of the SOX2 (0.29 ± 0.05, 0.21 ± 0.07, and 0.1 ± 0.03, respectively) and LIN28 proteins (0.06 ± 0.005, 0.045 ± 0.006, and 0.039 ± 0.006, respectively) were higher than those of VSMUi001-B. The PFFs expressed the lowest level of OCT4 (0.12 ± 0.006) and did not express SOX2 and LIN28 at all (Figure 3B and Supplementary Figure 4). After IF staining, only the VSMUi001-A and 5F-piPSC colonies were found to express proteomic pluripotency markers: OCT4, SOX2, NANOG, and LIN28 in their nuclei, and SSEA-1 on their surface. None of these colonies expressed SSEA-4. By contrast, a few colonies of VSMUi001-B expressed LIN28 and SSEA-1, and some also expressed OCT4 and SSEA-4 (Figure 4A). Additionally, the quantitative analysis of the IF staining under identical optical conditions confirmed that the mean fluorescence intensities of the immunoreactive pixels of OCT4, SOX2, LIN28, NANOG, and SSEA-1- were significantly brighter in the VSMUi001-A and 5TF-piPSC colonies than in the VSMUi001-B colonies. By contrast, that of SSEA-4 was significantly brighter in VSMUi001-B than in the other cell lines (Figure 4B). Furthermore, the flow cytometric analysis of the VSMUi001-A, VSMUi001-C, and VSMUi001-E cell lines revealed the percentage of OCT4^+^ cells to be greater than 89% (88 ± 1.63, 91.3 ± 1.50, 91.1 ± 1.78%, respectively) and that of SOX2^+^ cells to be greater than 80% (84.5 ± 1.49, 79.6 ± 2.15, 77.1 ± 2.29%, respectively). By contrast, the percentage of OCT4^+^ and SOX2^+^ VSMUi001-B cells was significantly lower (69.3 ± 1.11 and 4.9 ± 0.13%, respectively). Moreover, the percentage of LIN28^+^ cells was greater than 95% (96.9 ± 0.82, 97.9 ± 0.63, 96 ± 0.93%, respectively) and that of SSEA-1^+^ cells was 92% (92.1 ± 1.90, 92.7 ± 2.39, 92.1 ± 0.80%, respectively) in the VSMUi001-A, VSMUi001-C, and VSMUi001-E cell lines. By contrast, in the VSMUi001-B cell line, the percentages of LIN28^+^ and SSEA-1^+^ cells were also significantly lower (67.2 ± 1.27 and 0.5 ± 0.07%, respectively), whereas the percentage of SSEA-4^+^ cells (67.6 ± 0.67%) was significantly higher than that in the other cell lines (Figure 5).

### Spontaneous Cardiogenic Differentiation

**FIGURE 6:**
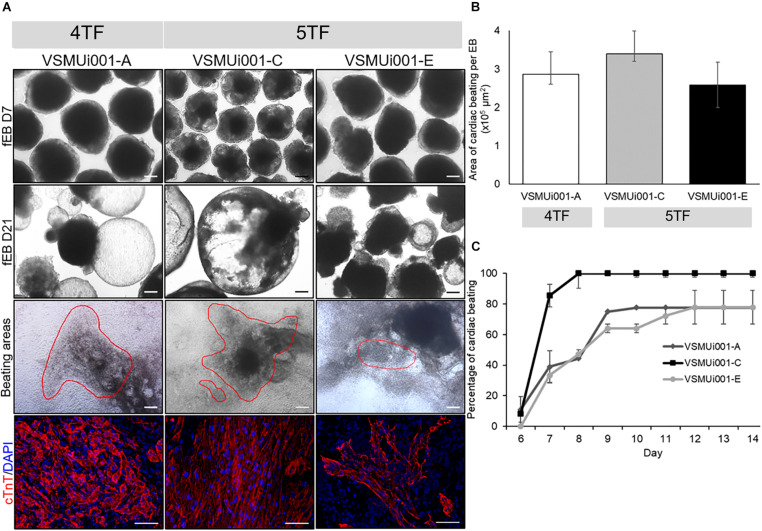
*In vitro* cardiogenic differentiation of various porcine-induced pluripotent stem cell lines. **(A)** Morphology of floating embryoid bodies (fEB) at days 7 and 21, area of cardiac beating (scale bar, 100 μm), and cTnT expression of VSMUi001-A, VSMUi001-C, and VSMUi001-E; scale bar, 50 μm. **(B)** Area of cardiac beating per embryoid body. **(C)** Percentage of spontaneous cardiac beating. 4TF, induction using OCT4, SOX2, KLF4, and C-MYC; 5TF, induction using OCT4, SOX2, KLF4, C-MYC, and LIN28; cTnT, cardiac troponin T.

All four piPSC lines were tested for EB formation to determine their differentiation capability *in vitro*. VSMUi001-A and the two 5TF-piPSC lines were able to form floating EBs, which were homogeneous in size and shape on day 7 and displayed cystic cavities on day 21 (Figure 6A). These three cell lines were therefore evaluated for spontaneous cardiogenic differentiation. However, because VSMUi001-B could not form floating EBs after day 3 and the ones that it did form in the first 3 days showed a limited ability to differentiate (Supplementary Figure 5), it was not evaluated for spontaneous differentiation. Spontaneously beating cardiomyocytes were visible on day 6, with the VSMUi001-C line being significantly faster than the other two cell lines in developing into these cardiac cells, displaying 100% of cardiac beating on day 8 and maintaining this percentage until day 14 (*P* < 0.01). VSMUi001-A and VSMUi001-E showed approximately 80% of cardiac beating on day 10 (78 ± 2.78%) and day 12 (77.78 ± 11.11%), respectively (Figure 6C). Moreover, the area of spontaneous cardiac beating appeared to be smaller for VSMUi001-E than for VSMUi001-A and VSMUi001-C (2.58 ± 0.59 × 10^5^, 2.86 ± 0.26 × 10^5^, and 3.39 ± 0.19 × 10^5^ μm^2^, respectively) (Figures 6A,B, and Supplementary Videos 1–3). All three piPSC lines expressed cardiac troponin T (cTnT), a specific cardiomyocyte marker (Figure 6A).

**FIGURE 7:**
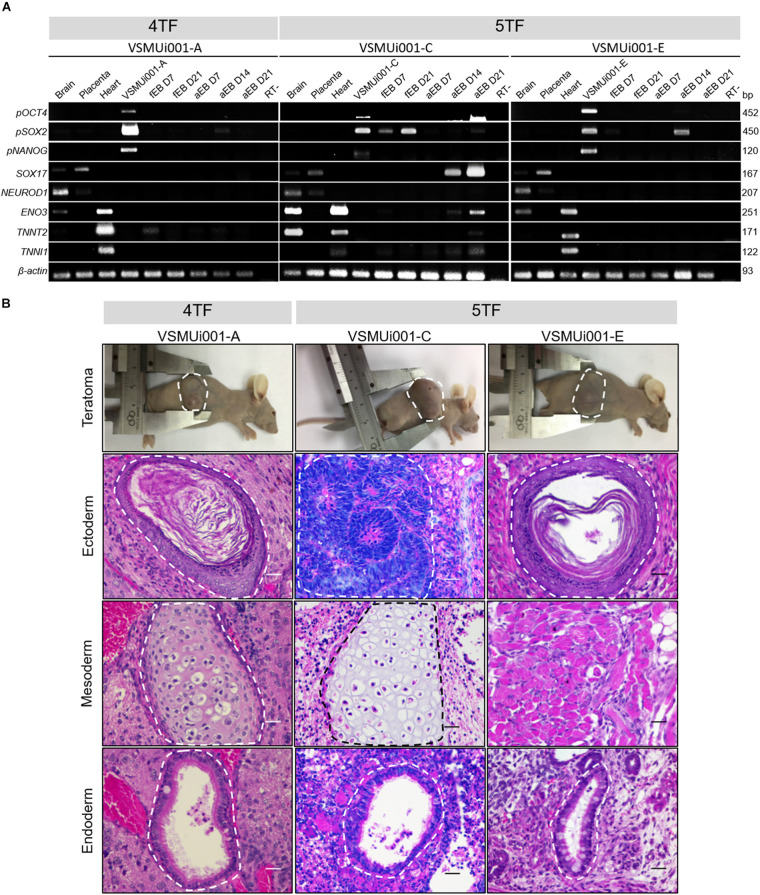
Expression of differentiation-related genes and *in vivo* differentiation of the various porcine-induced pluripotent stem cell (piPSC) lines. **(A)** Expression of differentiation genes specific for the three germ layers: *pOCT4*, *pSOX2*, and *pNANOG* (pluripotency genes); *SOX17* (endoderm); *NEUROD1* (ectoderm); and *ENO3, TNNT2* and *TNNI1* (mesoderm). fEB, floating embryoid body; aEB, adherent embryoid body. **(B)** Teratoma formation; all nude mice developed teratomas. Hematoxylin and eosin staining of the teratoma section generated by piPSCs showing a broad range of tissues of the three germ layers: keratinized squamous epithelia (VSMUi001-A and VSMUi001-E) and immature neuroepithelia forming rosettes (VSMUi001-C; ectoderm), cartilage (VSMUi001-A and VSMUi001-C), and skeletal muscle (VSMUi001-E; mesoderm), and respiratory-like epithelia (VSMUi001-A, VSMUi001-C, and VSMUi001-E; endoderm); scale bar, 25 μm. 4TF, induction using OCT4, SOX2, KLF4, and C-MYC; 5TF, induction using OCT4, SOX2, KLF4, C-MYC, and LIN28; *pOCT4*, endogenous porcine octamer-binding transcription factor 4; *pSOX2*, endogenous porcine SRY-box transcription factor 2; *pNANOG*, endogenous porcine Nanog homeobox; *SOX17*, SRY-box transcription factor 17; *NEUROD1*, neuronal differentiation 1; *ENO3*, enolase 3; *TNNT2*, troponin T2, cardiac type; TNNI1, troponin I1, slow skeletal type.

The abilities of VSMUi001-A, VSMUi001-C, and VSMUi001-E to express differentiation-related genes specific for the three germ layers were confirmed by RT-PCR. All three piPSC lines used for *in vitro* differentiation expressed the endogenous pluripotency markers *pOCT4*, *pSOX2*, and *pNANOG*. VSMUi001-A expressed the marker troponin T2, cardiac type (*TNNT2*) at day 7 and 21 of floating EB formation as well as at days 7, 14, and 21 of adherent EB formation. VSMUi001-C consistently expressed *pSOX2* at days 7 and 21 of floating EB formation and *pOCT4* at days 7 and 21 of floating EB formation and at days 14 and 21 of adherent EB formation. Moreover, VSMUi001-C also expressed differentiation-related gene markers for all three germ layers; namely, SRY-Box Transcription Factor 17 (*SOX17*) (endoderm) and Neuronal Differentiation 1 (*NEUROD1*) (ectoderm) at days 14 and 21 of adherent EB formation and Enolase 3 (*ENO3*), troponin T2, cardiac type (*TNNT2*) and troponin I1, slow skeletal type (*TNNI1*) (mesoderm) at days 7 and 21 of floating EB formation and days 7, 14, and 21 of adherent EB formation. By contrast, VSMUi001-E expressed only *pSOX2* at day 7 of floating EB formation and day 14 of adherent EB formation (Figure 7A and Supplementary Figure 6).

### Teratoma Formation

VSMUi001-A, VSMUi001-C, and VSMUi001-E were tested for their abilities to form teratomas as part of their *in vivo* differentiation capability. VSMUi001-B was excluded as it was undergoing apoptosis. At 35 days after piPSC injection, all nude mice developed solid tumors in each of the three germ layers, as shown by hematoxylin and eosin staining (Figure 7B). The teratomas contained a broad range of tissue types, including keratinized squamous epithelia and immature neuroepithelia forming rosettes (ectoderm), cartilage and skeletal muscle (mesoderm), and respiratory-like epithelia (endoderm) (Figure 7B).

## Discussion

In this study, we successfully generated piPSC colonies *via* the induction of PFFs by both four and five reprogramming factors using retroviral biotechnology. The reprogramming efficiency of the 4TF approach was quite low, resulting in only 0.002–2.7% AP-positive cells (Ruan et al., 2011; Gao et al., 2014; Setthawong et al., 2019). Furthermore, although the reprogramming efficiency of the 4TF system was higher than that of the 5TF system (0.33 versus 0.17%), its maintenance of self-renew and pluripotency (6.67%) was significantly lower than that of 5TF (100%). Taken together, this comparison study showed for the first time that the addition of Lin28 to OSKM TFs is more effective than OSKM alone for the following reasons: (1) 5TF can consistently establish self-renewal and pluripotency in all cell lines until passage 40 with 100% efficacy, whereas 4TF achieves this with 6.67% (1 out of 15 cell lines); and (2) further, all 5TF piPSC lines have the ability to differentiate into the three germ layers. Thus, the addition of LIN28 to 4TF was critical in enhancing the pluripotency and self-renewal capacity of piPSCs. In mammalian blastocysts, LIN28 is highly expressed in pluripotent cells of the inner cell mass and epiblast, which correlates with the intrinsic self-renewal ability of ESCs (Shyh-Chang and Daley, 2013). Moreover, LIN28 represses the maturation of *let-7* and prevents premature differentiation of the pluripotent cells of the inner cell mass and epiblast (Melton et al., 2010). Several studies have demonstrated that high LIN28 expression can improve the self-renewal capacity of ESCs *in vitro* (Bin et al., 2012; Shyh-Chang and Daley, 2013; Parisi et al., 2017; Mens and Ghanbari, 2018). Furthermore, models of LIN28 knockdown led to proliferative defects in mouse ESCs (Peng et al., 2011; Pan et al., 2018). LIN28, together with a cocktail of core reprogramming factors (OCT4, SOX2, and NANOG), was shown to promote hiPSC self-renewal (Hanna et al., 2009). In other studies, LIN28 promoted the self-renewal of mouse and human ESCs during reprogramming by upregulating numerous cell-cycle and cell growth regulators via *let-7* family repressors, such as Ras, Myc, high-mobility group A2 (Hmga2), insulin-like growth factor-2 mRNA-binding proteins (Igf2bps), and insulin–phosphoinositide 3-kinases (Pi3K)–mechanistic target of rapamycin (mTOR) pathways as well as other mRNAs encoding enzymes relevant to cell metabolism (Xu et al., 2009; Peng et al., 2011).

In this study, one of the 4TF-piPSC lines (VSMUi001-A) and two 5TF-piPSC lines revealed a typical mouse-like ESC morphology characterized by dome-shaped compact colonies with large nuclei and clear nucleoli and had SSEA-1 expression patterns similar to those reported in other piPSC studies (Cheng et al., 2012; Kwon et al., 2013; Zhang W. et al., 2015; Yuan et al., 2019) and our previous reports (Chakritbudsabong et al., 2017). As indicated in a previous report, LIN28 overexpression can enhance the derivation efficiency of miPSCs, where the loss of endogenous LIN28 facilitated the conversion of miPSCs from naïve to primed pluripotent cells through the induction of the FGF/activin signaling pathway (Zhang J. et al., 2016). Most of the 4TF-piPSCs exhibited low levels of SSEA-1 and LIN28, whereas they expressed high levels of SSEA-4. However, piPSCs have been shown to display varied expression patterns of the surface pluripotency markers SSEA-1, SSEA-3, and SSEA-4 (Ruan et al., 2011; Liu et al., 2012; Gao et al., 2014; Chakritbudsabong et al., 2017). This is of concern because it shows the high heterogeneity of the cells being generated by the different induction and maintenance methods (Koh and Piedrahita, 2014). Although the 4TF-piPSC line VSMUi001-A and the two 5TF-piPSC lines required both bFGF and LIF for maintaining pluripotency, the surface of all piPSCs expressed SSEA-1. Moreover, VSMUi001-A could express a high level of LIN28 as a result of the activation of core pluripotency TFs. OCT4, SOX2, and NANOG could regulate the transcription of LIN28 in mammalian ESCs (Marson et al., 2008) and SOX2 has been found to be the most critical factor for regulating LIN28A expression during iPSC reprogramming (Buganim et al., 2012). SOX2 is closely related to LIN28A in pluripotency because it binds directly to LIN28A to form a nuclear protein–protein complex (Cox et al., 2010).

In human and mouse iPSCs, retroviral vectors are transcriptionally silent as the endogenous genes maintain the pluripotent during iPSC induction, known as a fully reprogrammed iPSC state. On the contrary, partially reprogrammed iPSCs express both the viral transgenes and endogenous pluripotency genes (Maherali et al., 2007; Okita et al., 2007; Wernig et al., 2007; Hotta and Ellis, 2008). Substantial obstacles remain in the establishment of piPSCs, including the lack of transgene silencing of plasmid DNA integrated into the genome and the inability of cells to proliferate in the absence of transgene expression. These challenges suggest that reprogramming of these piPSCs is not fully complete (Esteban et al., 2009; Cheng et al., 2012; Liu et al., 2012; Zhang W. et al., 2015). Similarly, all piPSC lines in our study do not fully undergo reprogramming, expressing viral transgenes and endogenous pluripotency genes from the retrovirus-mediated gene delivery approach. Up to now, only one study in the literature has described silenced retroviral transgene expression (Zhang S. et al., 2015). To try to overcome this issue, researchers have made efforts to establish transgene-free piPSCs using integration-free reprogramming approaches such as episomal vectors (Telugu et al., 2010; Du et al., 2015; Li et al., 2018; Yuan et al., 2019). However, from these latter four reports, three demonstrated genome integration of the plasmid DNA into the piPSC genome (Telugu et al., 2010; Du et al., 2015; Yuan et al., 2019), and one described persistent exogenous gene expression in most piPSC lines with partial reprogramming (Li et al., 2018). Regarding reprogramming techniques using Sendai-viral vectors, only one study showed integration-free reprogramming though this group still reported strong exogenous transgene (*hsOCT4*) expression (Congras et al., 2016). Our reprogramming approach using monocistronic vectors can cause random expression of TFs in cells and partial reprogramming of piPSCs, which may potentially lead to partial reprogramming of piPSCs (Esteban et al., 2009). This is a limitation that can be overcome by using polycistronic vectors in future studies. Recently, polycistronic vectors have recently enabled the simultaneous delivery and expression of numerous genes, as well as the efficient co-expression of TFs (Shaimardanova et al., 2019).

Numerous previous studies have demonstrated that human and mouse TFs are capable of reprogramming porcine somatic cells to form piPSCs (Ma et al., 2014; Fukuda et al., 2017; Setthawong et al., 2019; Yuan et al., 2019). Similarly, in our study, piPSCs were successfully generated using factors associated with human reprogramming. Additionally, a previous report observed no differences in the morphology, AP staining, or expression of pluripotency markers between piPSC generated from mouse or human TFs (Esteban et al., 2009). Contrary to other studies, only piPSCs reprogrammed with human TFs were capable of completely reprogramming porcine cells to piPSCs (Zhang S. et al., 2015) and developing live chimeric offspring (West et al., 2010). At the moment, the researchers are using porcine TFs to generate piPSCs. The reprogramming of piPSCs with porcine TFs has the potential to generate both embryonic and extraembryonic cells (Gao et al., 2019; Xu et al., 2019). In terms of DNA methylation, the previous study indicated that while piPSCs had a higher proportion of methylation at the OCT4 promoter, blastocyst and PFFs had a low rate of methylation. It demonstrated that the OCT4 gene was substantially expressed in piPSCs when compared to blastocysts and PFFs (Fujishiro et al., 2013). In this study, the methylation patterns in porcine OCT4 promoter region were not established using bisulfite sequencing as published previously (Choi et al., 2016) and require further assessment.

For *in vitro* differentiation, VSMUi001-A and the two 5TF-piPSC lines had the capability to differentiate into the three embryonic germ layers and toward a specific cardiac lineage, during which the *SOX2* gene was continuously upregulated. SOX2 is expressed not only in pluripotent stem cells but also in neural stem cells, which are maintained during the expansion of neural precursors throughout the development of the central nervous system and into adulthood (Pevny and Nicolis, 2010; Mercurio et al., 2019). Hence, the expression of SOX2 in the EBs as well as the *in vitro* spontaneous cardiogenic differentiation suggested that our piPSCs could also differentiate toward the neural lineage. For *in vivo* differentiation, teratoma development was slightly faster after 5TF-piPSC injection than after 4TF-piPSC injection. According to another study, high LIN28 expression generates higher-grade teratomas, whereas LIN28 knockdown induces smaller tumors (West et al., 2009). The chimera formation assay is the gold standard for determining the pluripotency of stem cells *in vivo*. Gao et al. (2019) generated extended pluripotent stem cells (EPSCs) that differentiate into both embryonic and extra-embryonic tissue in the pig blastocyst. Until now, this was the only report describing the production of a live chimeric pig in which piPSCs were discovered in the ears and tails (West et al., 2010).

Porcine iPSCs are a promising cellular source for the three-dimensional reconstruction of human-like organoids mimicking organ/system homeostasis or diseases, especially those related to the cardiovascular system. Cardiomyocytes derived from piPSCs can fulfill the application of PSCs in cardiac regenerative medicine and especially provide a porcine model for the evaluation of the efficiency, safety, and side effects of iPSC transplants (Zhou et al., 2012, 2014). Gu et al. (2012) revealed that piPSC-derived endothelial cells could induce neovascularization and important paracrine factors for myocardial healing, making them an effective treatment for myocardial infarction. However, there are not many studies that have reported the differentiation of piPSCs into cardiomyocytes (Montserrat et al., 2011; Chakritbudsabong et al., 2017). In this study, the cardiac lineage markers (TNNT2 and TNNI1) were visible since day 7 of floating EB formation. The three piPSC lines could differentiate into cells with mature cardiac phenotypes that expressed cTnT and cardiac contractile protein and displayed a high percentage of spontaneous cardiac beating. Xiang et al. (2019) have shown that LIN28 plays a critical role in the tumor necrosis factor receptor-2 (TNFR2)-mediated differentiation of hiPSC-derived cardiac stem cells by inducing the expression of TNFR2, which is usually found in mature cardiomyocytes, vascular endothelial cells, and hematopoietic cells. Conversely, LIN28 inhibition significantly reduced cardiac stem cell differentiation and activation (Xiang et al., 2019). In summary, our study demonstrated an effective method for inducing the spontaneous differentiation of piPSCs into cardiomyocytes *via* EB formation. In ongoing research, we aim to develop a robust biotechnology culture platform to (1) increase the number of piPSC-derived cardiomyocytes for future scalability, (2) develop organ-on-dish cardiac models for studying cardiovascular diseases and screening novel compounds for drug discovery; and (3) study the efficiency of piPSC-derived cardiomyocytes for use as a swine model for studying human cardiovascular diseases. Besides, these piPSCs are an innovative tool that can be used for study of disease pathologies and drug discovery for veterinary science.

## Conclusion

The addition of LIN28 to the 4TF-induced reprogramming of piPSCs promoted the long-term maintenance of piPSC self-renewal and pluripotency and enhanced both the *in vitro* and *in vivo* differentiation capabilities of the cells toward all three embryonic germ layers and the cardiac lineage. Additionally, the spontaneous beating of the differentiated cardiomyocytes was augmented under the 5TF induction approach. This study proves that the vital role of LIN28 in the induction of pluripotency applies not only to hiPSCs but to piPSCs as well, thereby resolving the current challenges faced over the long-term maintenance of piPSC self-renewal and pluripotency. Importantly, our findings allow for the efficient scale-up of piPSC-derived cardiomyocytes for application in research studies on cardiovascular diseases and treatments. Further, the application of piPSCs and their differentiated cells are also great valuable in veterinary research.

## Data Availability Statement

The original contributions presented in the study are included in the article/Supplementary Material, further inquiries can be directed to the corresponding author.

## Ethics Statement

The animal study was reviewed and approved by the Institutional Animal Care and Use Committee at the Faculty of Veterinary Science, Mahidol University, Thailand (Approval ID: VSMU-2012-57).

## Author Contributions

WC, JF, and SR designed the project and wrote the manuscript. WC, SC, and SR carried out most experiments, including the establishment and characterization of piPSC lines. LS performed molecular analysis. WC and SP conducted G-banding karyotype analysis. WC and PS performed immunofluorescence staining. WC and DG performed flow cytometry analysis. TT constructed pMXs plasmids. AD, JF, and SR revised the manuscript. All authors read and approved the final version submitted.

## Conflict of Interest

AD is employed by BioTalentum Ltd., Hungary. The remaining authors declare that the research was conducted in the absence of any commercial or financial relationships that could be construed as a potential conflict of interest.
