# Supplementary material for: Exogenous LIN28 Is Required for the Maintenance of Self-Renewal and Pluripotency in Presumptive Porcine-Induced Pluripotent Stem Cells
Source: Front Cell Dev Biol. 2021 Jul 20;9:709286. doi: 10.3389/fcell.2021.709286 (PMC8329718; doi:10.3389/fcell.2021.709286)
Supplement: Supplementary file 1 [file Data_Sheet_1.pdf]

Supplementary Material

1 Supplementary Figures and Tables

Supplementary Table 1. Antibodies used for immunofluorescence

Markers	Antibodies	Dilution	Companies	Cat #
Pluripotency	Goat anti-OCT4	1:200	Santa Cruz Biotechnology	sc-8628
	Rabbit anti-SOX2	1:100	Santa Cruz Biotechnology	sc-20088
	Goat anti-NANOG	1:100	R&D system	AF 1997
	Mouse anti-SSEA-1	1:100	Santa Cruz Biotechnology	sc-101462
	Mouse anti-SSEA-4	1:100	Santa Cruz Biotechnology	sc-21704
	Rabbit anti-LIN28	1:200	Abcam	AB63740
In vitro differentiation	Mouse anti-cTnT	1:100	Santa Cruz Biotechnology	sc-20025
Secondary antibodies	Alexa Fluor 488 donkey anti-goat IgG	1:1,000	Thermo Fisher Scientific	A-11055
	Alexa Fluor 488 donkey anti-rabbit IgG	1:1,000	Thermo Fisher Scientific	A-21206
	Alexa Fluor 594 donkey anti-rabbit IgG	1:1,000	Thermo Fisher Scientific	A-21207
	Alexa Fluor 594 donkey anti-mouse IgG	1:1,000	Thermo Fisher Scientific	A-21203
	Goat anti-mouse IgM-PE	1:1,000	Santa Cruz Biotechnology	sc-3768

Supplementary Table 2. Primers used for reverse transcription polymerase chain reaction

Genes	Primer sequence (5′-3′) Forward	Reverse	Product size (bp)
**Exogenous genes**			
hOCT4	GTT GCT CTC CAC CCC GAC TCC TGC TTC	GAG AAC CGA GTG AGA GGC AAC	250
hSOX2	CCC CTG TGG TTA CCT CTT CCT CC	TGC CGT TAA TGG CCG TGC C	176
hKLF4	GGC TGA TGG GCA AGT TCG	CTG ATC GGG CAG GAA GGA T	400
hC-MYC	GCA GCG ACT CTG AGG AGG AAC AA	TTT TCC TTA CGC ACA AGA GTT CCG T	581
hLIN28	TCA GCC GAC GAC CAT GGG CT	CCA TGT GCA GCT TAC TCT GGT GCA C	250
**Endogenous gene**			
pOCT4	ACA AGG AGA AGC TGG AGC CG	CGC GGA CCA CAT CCT TCT CT	752
pSOX2	GGT TAC CTC TTC TTC CCA CTC CA	CAA AAA TAG TCC CCC CAA AAG AAG	450
pNANOG	TCT GTG TCA GTT TGA GGG ACA GG	AAC AAG TAA AGC CTC CCT ATC CCA	120
pLIN28	CAG AGT AAG CTG CAC ATG GAG G	GTA GGC TGG CTT TCC CTG TG	325
**Endoderm genes**			
SOX17	CGC ACG GAG TTT GAA CAA TA	CAG ACG TCG GGG TAG TTA CAG	167
**Ectoderm genes**			
NEUROD1	GAC TTG CGT TCA GGC AAA AGC	GGG CGAC TGG TAA GAG TAG G	207
**Mesoderm and cardiac differentiation marker**			
ENO3	TCT GTG ACT GAA TCT ATC CAG G	CTT TGG GTT ACG GAA CTT GCG	251
TNNT2	GAC GGA GCG TAA GAG TGG	CAG GTC AAA CTT CTC CGC C	171
TNNI1	AGA GAA AAC CCA AGA TCA CTG	CAG GTA GCG AGC CTT CTC A	122

**Housekeeping gene**			
β-actin	CGG GAC CTG ACT GAC TAC CTC	CCT TAA TGT CAC GCA CGA TTT CC	93

Supplementary Table 3. The efficiency of piPSCs

Induced system	Initial transfected cells	AP-positive colonies (%)*	No. of picked up colonies	No. of iPSC lines up to P20 (%)**	No. of iPSC lines up to P40 (%)***
4TF	4,500	15 (0.33)	15	2 (13.33)	1 (6.67)
5TF	1,800	3 (0.17)	3	3 (100)	3 (100)

* The percentage of AP-positive colonies was calculated as the number of AP positive colonies divided by the total number of transfected cells

** The percentage of iPSC lines was calculated as the number of iPSC lines (up to P20) divided by the total number of picked up colonies

*** The percentage of iPSC lines was calculated as the number of iPSC lines (up to P40) divided by the total number of picked up colonies

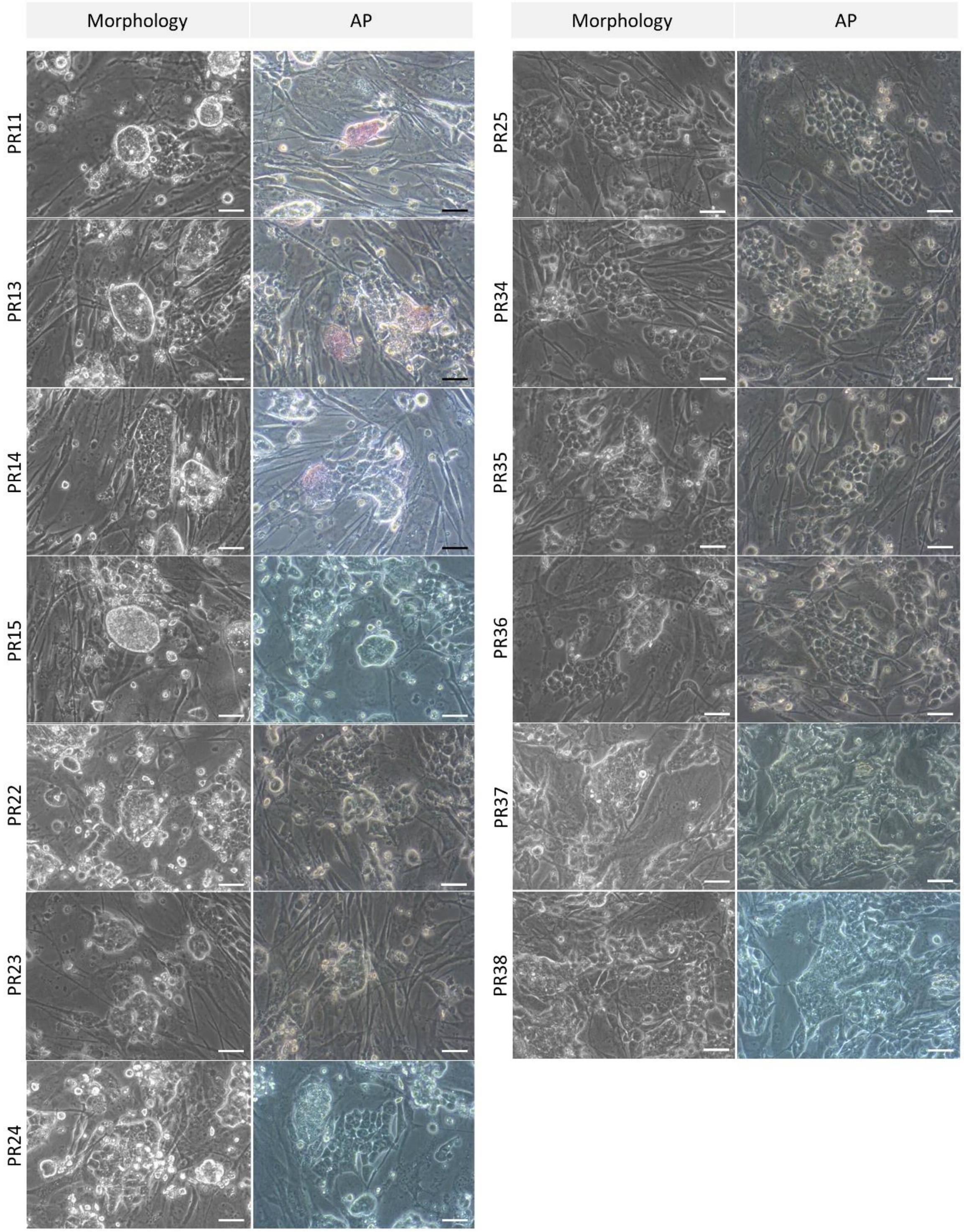

Supplementary Figure 1. The morphology and AP staining of 4TF-piPSC colonies at P5 except VSMUi001-A and VSMUi001-B; scale bar, 20 μm.

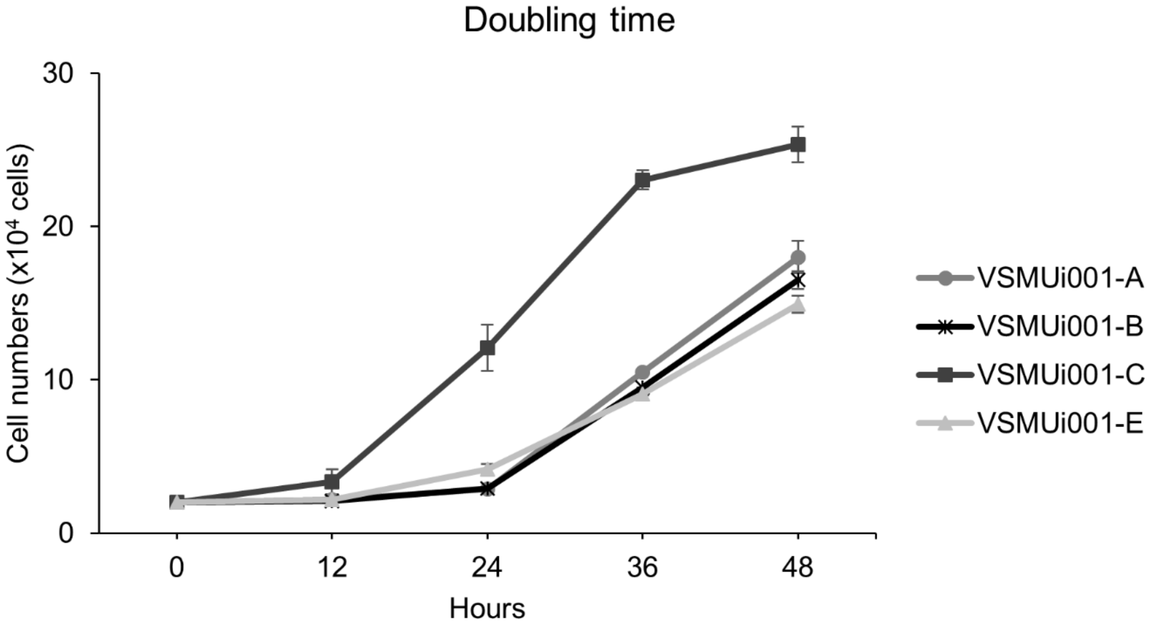

Supplementary Figure 2. Population doubling time of 4TF-piPSC and 5TF-piPSC cell lines

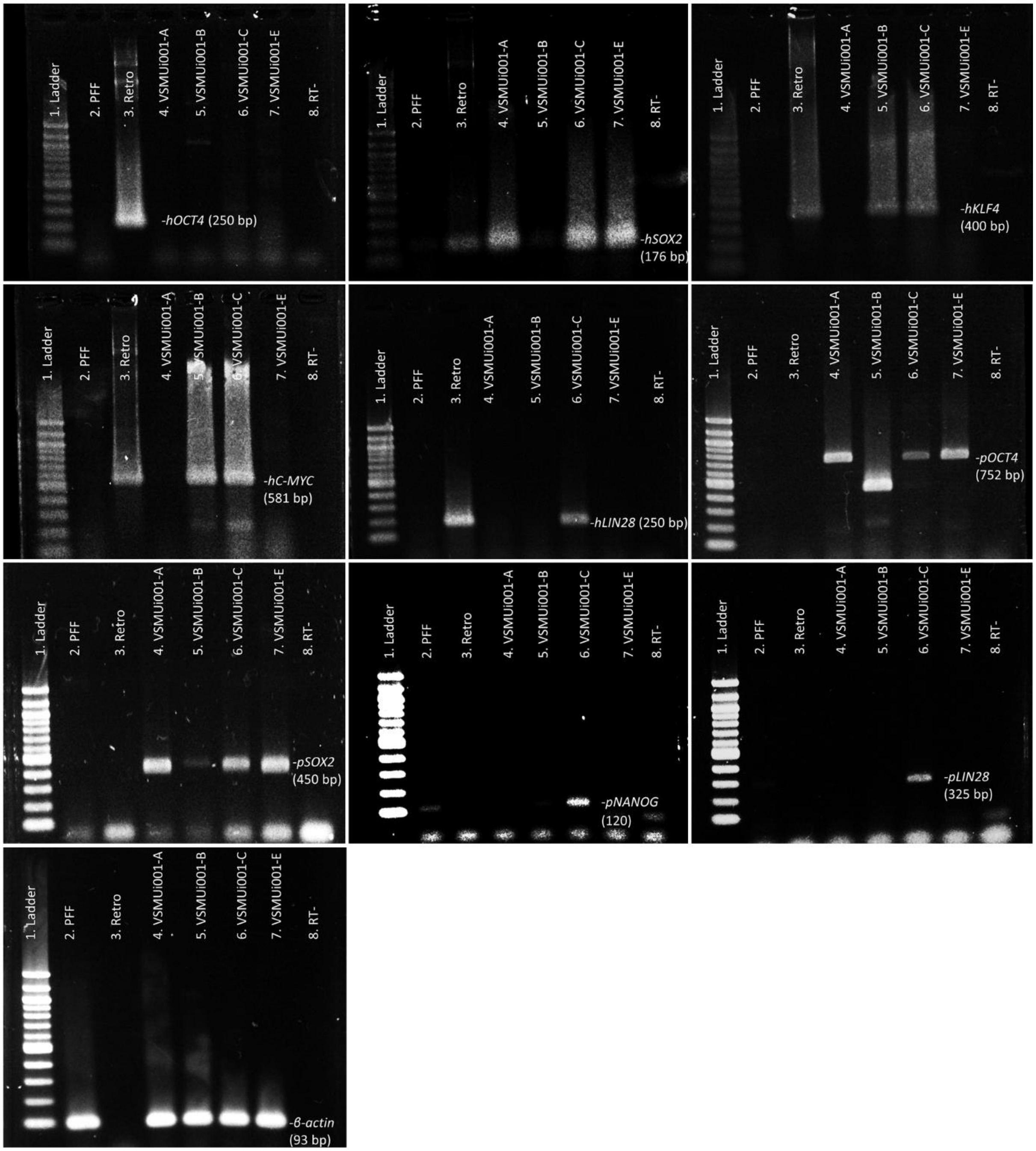

Supplementary Figure 3. Original RT-PCR of Figure 3A

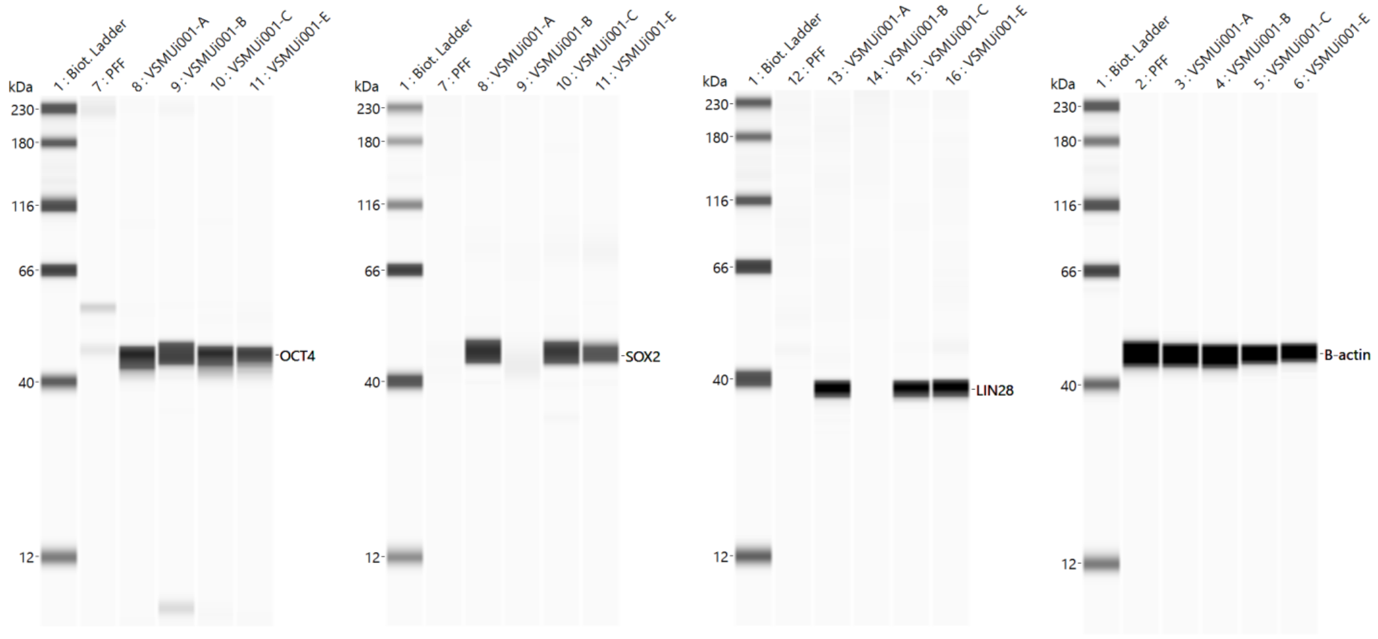

Supplementary Figure 4. Original blots of Western blot of Figure 3B

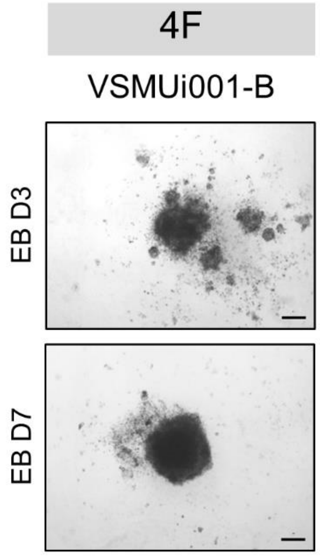

Supplementary Figure 5. EB formation of VSMUi001-B 4TF-piPSC at days 3 and 7; scale bar, 100 μm

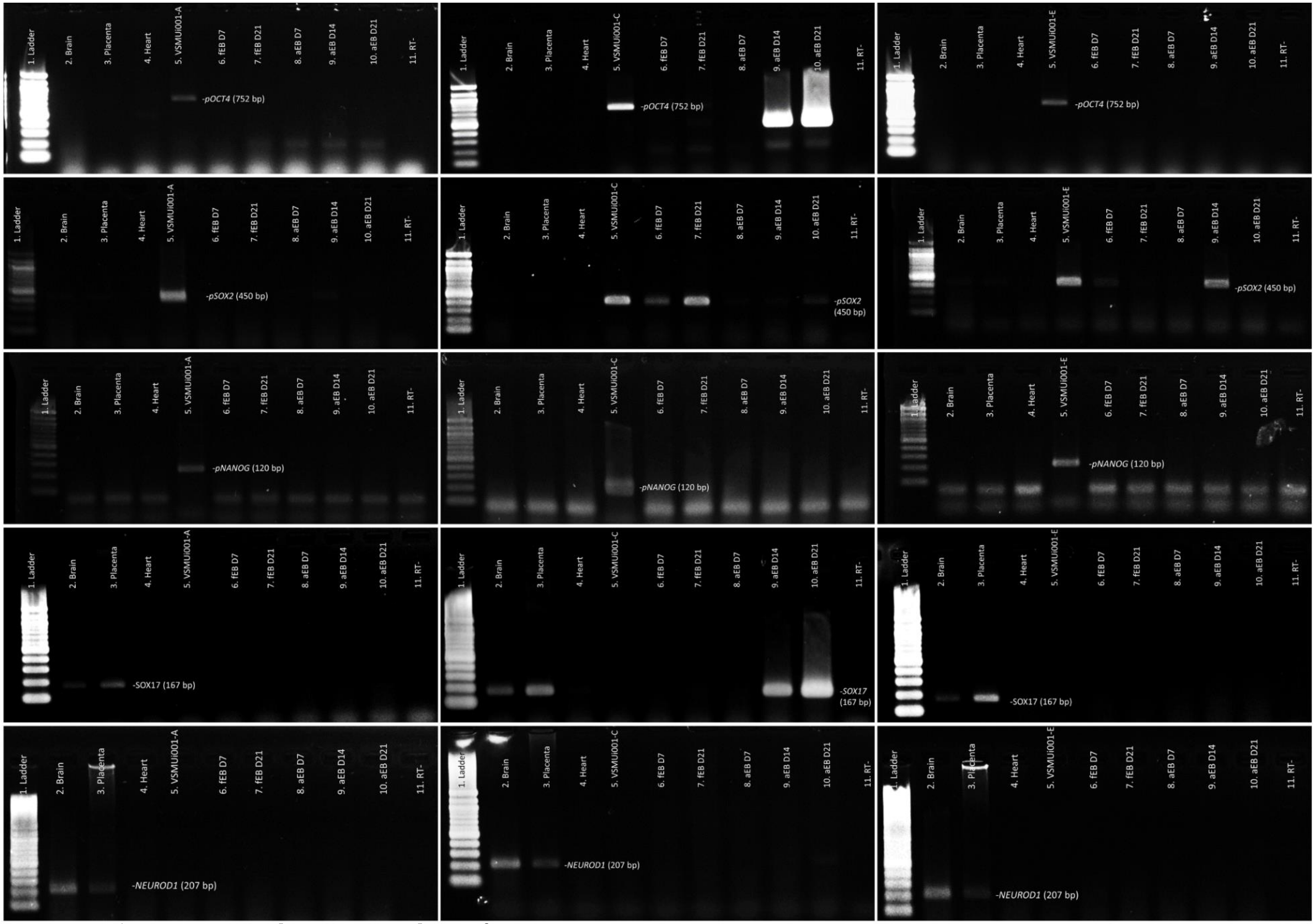

Supplementary Figure 6. Original RT-PCR analysis of Figure 7A

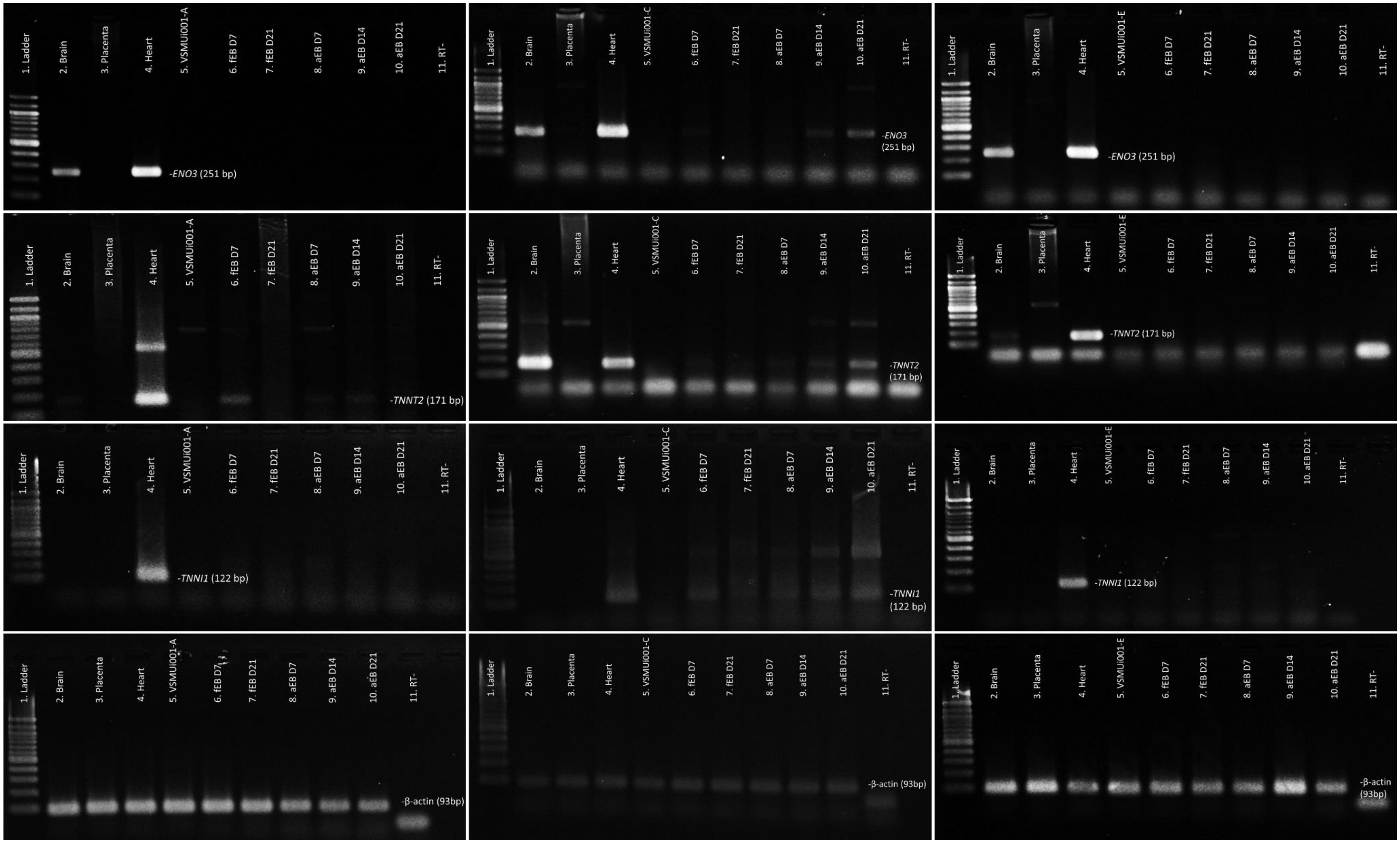

Supplementary Figure 6. Original RT-PCR analysis of Figure 7A (Cont)

Supplementary Video 1. Spontaneous cardiac beating of VSMUi001-A 4TF-piPSC (40× magnification), Related to Figure 6A

Supplementary Video 2. Spontaneous cardiac beating of VSMUi001-C 5TF-piPSC (40× magnification), Related to Figure 6A

Supplementary Video 3. Spontaneous cardiac beating of VSMUi001-E 5TF-piPSC (40× magnification), Related to Figure 6A
